# Association of Nrf2-encoding *NFE2L2 *haplotypes with Parkinson's disease

**DOI:** 10.1186/1471-2350-11-36

**Published:** 2010-03-02

**Authors:** Malin von Otter, Sara Landgren, Staffan Nilsson, Dragana Celojevic, Petra Bergström, Anna Håkansson, Hans Nissbrandt, Marek Drozdzik, Monika Bialecka, Mateusz Kurzawski, Kaj Blennow, Michael Nilsson, Ola Hammarsten, Henrik Zetterberg

**Affiliations:** 1 Institute of Neuroscience and Physiology, Department of Psychiatry and Neurochemistry, The Sahlgrenska Academy at University of Gothenburg, Blå stråket 15, 413 45 Gothenburg, Sweden; 2 Institute of Neuroscience and Physiology, Department of Pharmacology, The Sahlgrenska Academy at University of Gothenburg, Box 431, 405 30 Gothenburg, Sweden; 3 Institute of Mathematical Sciences, Department of Mathematical Statistics, Chalmers University of Technology, Chalmers tvärgata 3, 412 96 Gothenburg, Sweden; 4 Institute of Biomedicine, Department of Clinical Chemistry and Transfusion Medicine, The Sahlgrenska Academy at University of Gothenburg, Bruna stråket 16, 413 45 Gothenburg, Sweden; 5 Department of Pharmacology, Pomeranian Medical University, Powstancow Wlkp 72, Szczecin 70-111, Poland; 6 Institute of Neuroscience and Physiology, Center for Brain Repair and Rehabilitation, The Sahlgrenska Academy at University of Gothenburg, Per Dubbsgatan 14, 413 45, Gothenburg, Sweden

## Abstract

**Background:**

Oxidative stress is heavily implicated in the pathogenic process of Parkinson's disease. Varying capacity to detoxify radical oxygen species through induction of phase II antioxidant enzymes in substantia nigra may influence disease risk. Here, we hypothesize that variation in *NFE2L2 *and *KEAP1*, the genes encoding the two major regulators of the phase II response, may affect the risk of Parkinson's disease.

**Methods:**

The study included a Swedish discovery case-control material (165 cases and 190 controls) and a Polish replication case-control material (192 cases and 192 controls). Eight tag single nucleotide polymorphisms representing the variation in *NFE2L2 *and three representing the variation in *KEAP1 *were chosen using HapMap data and were genotyped using TaqMan Allelic Discrimination.

**Results:**

We identified a protective *NFE2L2 *haplotype in both of our European case-control materials. Each haplotype allele was associated with five years later age at onset of the disease (p = 0.001) in the Swedish material, and decreased risk of PD (p = 2 × 10^-6^), with an odds ratio of 0.4 (95% CI 0.3-0.6) for heterozygous and 0.2 (95% CI 0.1-0.4) for homozygous carriers, in the Polish material. The identified haplotype includes a functional promoter haplotype previously associated with high transcriptional activity. Genetic variation in *KEAP1 *did not show any associations.

**Conclusion:**

These data suggest that variation in *NFE2L2 *modifies the Parkinson's disease process and provide another link between oxidative stress and neurodegeneration.

## Background

Oxidative stress has been implicated as a major contributing factor in neurodegenerative diseases in general and Parkinson's disease (PD) in particular [1]. Cellular responses to oxidative stress are major determinants of disease susceptibility and aging, particularly in tissues that are sensitive to oxidative stress, such as the central nervous system [2,3]. In PD brain specimens, signs of oxidative stress are especially prominent in the substantia nigra. This may be the result of combined presence of a high dopamine metabolism generating reactive oxygen species (ROS), low levels of antioxidant glutathione and increased levels of iron catalyzing ROS formation [4]. Furthermore, genetic aberrations in oxidative responses may cause neurodegenerative diseases. Examples include mutations in *SOD1 *(encoding superoxide dismutase 1) that cause amyotrophic lateral sclerosis [5] and loss of function mutations in *DJ-1 *(encoding Parkinson disease protein 7) that leads to early onset PD with high penetrance [6].

Nuclear factor-erythroid 2 (NF-E2)-related factor 2 (Nrf2) is a member of the cap 'n' collar family of basic leucine zipper transcription factors that regulate the expression of many antioxidant pathway genes in the so-called phase II response [7]. Nrf2 is maintained at basal levels in cells by binding to its inhibitor protein, Kelch-like erythroid-cell-derived protein with CNC homology (ECH)-associated protein 1 (Keap1) [8,9]. Keap1 is a BTB (Broad complex, Tramtrack, Bric-a-Brac) domain-containing protein [9] that targets Nrf2 for ubiquitination by Cul3/Roc-1, leading to its constitutive degradation [10-13]. Upon exposure to oxidative stress, xenobiotics, or electrophilic metabolites of phase I enzymes, repression of Nrf2 by Keap1 ubiquitination is disrupted and newly produced Nrf2 enters the nucleus [14]. There, it forms heterodimers with other transcription regulators, such as small Maf proteins, and induces the expression of antioxidant phase II genes through interaction with the antioxidant responsive element (ARE) in the promoter of these genes [15,16]. Binding of Nrf2 to ARE drives the expression of phase II enzymes, such as NQO1 [NAD(P)H dehydrogenase (quinine) 1] and HO-1 (Heme oxygenase 1), that generate antioxidant molecules, such as glutathione [17,18]. Nrf2 has been shown to protect neurons from acute injury in culture [19-21] and *in vivo *[22]. Furthermore, upregulation of Nrf2 activity in astrocytes delays motor neuron degeneration in a mouse model of familial amyotrophic lateral sclerosis [23].

Apart from the undisputed involvement of oxidative stress in the PD process [1] there are additional and more specific links between Nrf2 function and PD. First, nuclear localization of Nrf2 is induced in PD-affected substantia nigra, even though the response appears insufficient to protect neurons from degeneration [24]. Second, treatment of nigrostriatal cultures with Nrf2 activators protects from oxidative stress-induced loss of dopaminergic cells [25]. Third, a recently discovered function of *DJ-1 *is to stabilize Nrf2 by preventing its interaction with Keap1 [26]. Fourth, recent and quite striking data show that induced expression of Nrf2 in brains of transgenic mice protects from MPTP (1-methyl-4-phenyl-1,2,3,6-tetrahydropyridine)-caused damage to the nigrostriatal dopaminergic pathway as seen in PD [27].

Despite these extensive preclinical data, no association has as yet been demonstrated between *NFE2L2 *and *KEAP1*, the Nrf2 or Keap1 encoding genes, and neurodegenerative disease [28]. Here, we have, for the first time, performed a complete haplotype analysis of the *NFE2L2 *and *KEAP1 *genes in relation to risk of PD in two independent case-control materials. We found strong protective effects of an *NFE2L2 *haplotype in two independent case-control materials, indicating that varying efficiency in the oxidative protection by Nrf2 may influence PD pathogenesis.

## Methods

### Case-control materials

The Swedish discovery material consisted of 165 PD cases and 190 age-matched controls. All individuals were of Caucasian origin. The cases fulfilled the Parkinson's Disease Society Brain Bank criteria for idiopathic PD [29], except for that the presence of more than one relative with PD was not considered an exclusion criterion. PD cases with an age at onset (AAO) of <50 years were screened to exclude that they were carriers of recognized PD-causing mutations in the *DJ-1*, *Parkin*, *PINK1 *and *LRRK2 *genes [30,31]. Demographic characteristics are given in table 1.

**Table 1:** Demographic characteristics of PD cases and controls

Parameter	Parkinson	Control	p-value^1^
***Swedish cases and controls***			
Number of subjects	165	190	
Age at sampling (years)	68.2 ± 8.8	69.1 ± 9.3	0.698
Age at onset (years)	59.0 ± 10.2	---	---
Sex (Female)	71 (43.0)	120 (63.2)	<0.001
(Male)	94 (57.0)	70 (36.8)	
Current smoker	9 (8.7)	13 (8.3)	0.897
Ever smoked	38 (36.9)	81 (51.6)	0.020
Parkinson's disease in family	40 (24.8)	18 (9.5)	<0.001
Alzheimer's disease in family	7 (4.3)	8 (4.3)	0.976
***Polish cases and controls***			
Number of subjects	192	192	
Age at sampling (years)	63.7 ± 10.9	72.9 ± 9.9	<0.001
Age at onset (years)	55.2 ± 10.9	---	---
Sex (Female)	75 (39.1)	75 (39.1)	1.000
(Male)	117 (60.9)	117 (60.9)	

Data are presented as absolute numbers (%) or mean ± SD. ^1^p-values are calculated with χ^2^-test for categorical parameters and Mann-Whitney U test for continuous parameters.

The Polish replication material consisted of 192 PD cases and 192 sex-matched controls. Controls were chosen to be of as high age as possible when included in the study to minimize the number of controls developing PD later in life. All individuals were of Caucasian origin and had no familial aggregation of PD. Demographic characteristics are given in table 1.

### Tag SNP selection

**Figure 1:**
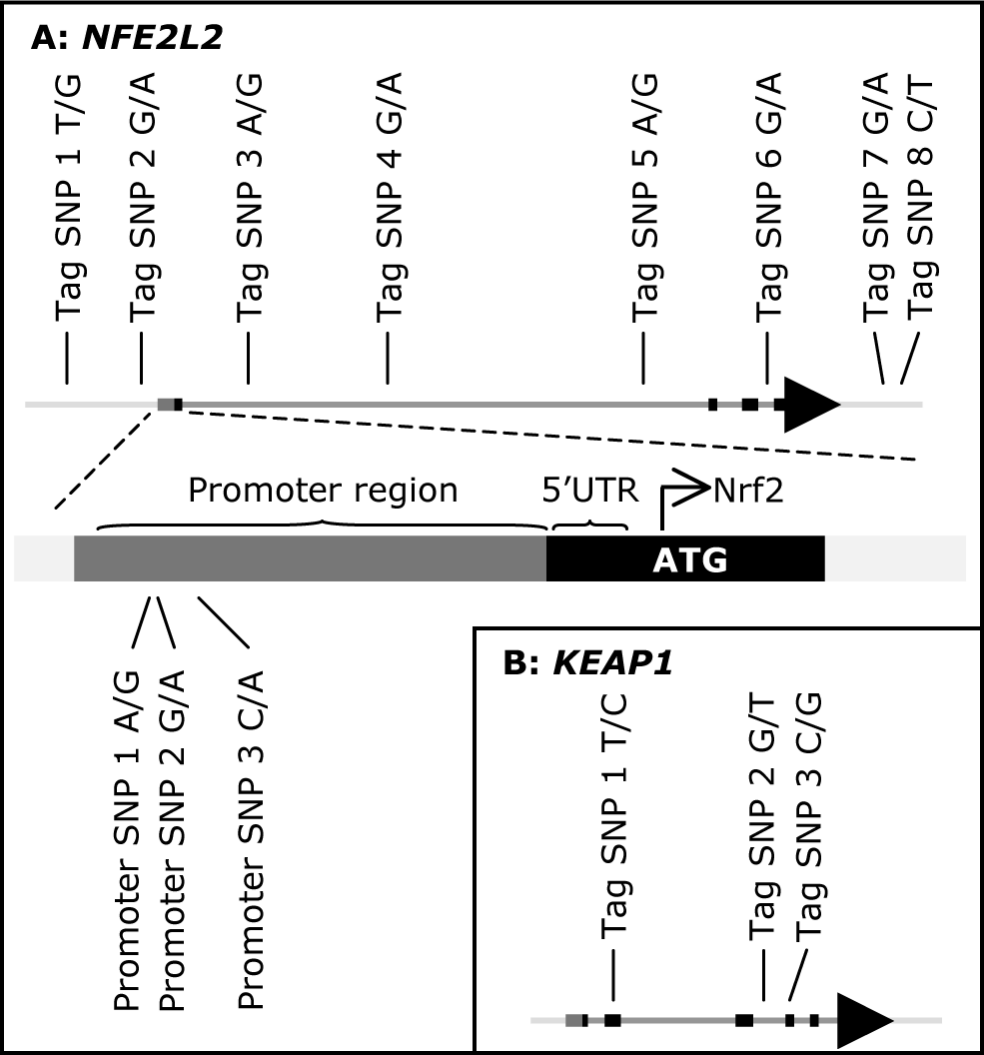
**Schematic overview of the *NFE2L2 *(panel A) and *KEAP1 *(panel B) genes and the tag SNPs used in the study (for rs-numbers see table 2)**. Thick black lines indicate exons. The promoter region of *NFE2L2 *is detailed in panel A.

Single nucleotide polymorphism (SNP) genotyping data covering *NFE2L2 *and *KEAP1 *for the European material CEU (Utah residents with ancestry from northern and western Europe) were downloaded from the International Haplotype Mapping Project web site http://www.hapmap.org[32] and processed in the Haploview software [33]. Linkage disequilibrium (LD) blocks were constructed according to Gabriel *et al*. [34] and tag SNPs were assigned using the tagger function [33]. A minor allele frequency of ≥ 5% and pair wise tagging with a minimum r^2 ^of 0.80 were applied to capture the common variations within the blocks covering *NFE2L2 *and *KEAP1*. The common genetic variation of *NFE2L2 *was tagged for by eight tag SNPs: rs16865105, rs7557529, rs2886161, rs1806649, rs2001350, rs10183914, rs2706110 and rs13035806, and *KEAP1 *by three tag SNPs: rs1048290, rs11085735 and rs1048287 (figure 1, table 2).

**Table 2:** Overview of location and type of the SNPs studied

SNP	rs-ID	Genome Position	Alleles	Gene location	SNP type	TaqMan Assay
***NFE2L2***		***Chr:2(-)***				
1	rs16865105	177844875	T>G	5'-region	---	C_33808341_10
2	rs7557529	177843343	G>A	5'-region	---	C__436313_10
P1	rs35652124	177838319	A>G	Promoter (-653)	Regulatory^1^	Sequencing^2^
P2	rs6706649	177838317	G>A	Promoter (-651)	Regulatory^1^	Sequencing^2^
P3	rs6721961	177838317	C>A	Promoter (-617)	Regulatory^1^	Sequencing^2^
3	rs2886161	177836085	A>G	Intron 1	---	C__351881_10
4	rs1806649	177826398	G>A	Intron 1	---	C_11634983_10
5	rs2001350	177808671	A>G	Intron 1	---	C_11634985_10
6	rs10183914	177805912	G>A	Intron 3	---	C__157561_10
7	rs2706110	177800408	G>A	3'-region	---	C_11745133_10
8	rs13035806	177800068	C>T	3'-region	---	C_11745134_10
***KEAP1***		***Chr:19(-)***				
1	rs1048287	10471236	T>C	Exon 2	Synonymous	C___9323068_10
2	rs11085735	10463180	G>T	Intron 3	---	Custom^3^
3	rs1048290	10461442	C>G	Exon 4	Synonymous	C___9323035_1_

Presented are SNPs numbered according to location on the gene. Genome positions were obtained from the HapMap Genome Browser (Phase 1 & 2 - full dataset) at the International Haplotype Mapping Project web site http://www.hapmap.org. Alleles are given according to the sense sequence of the gene. ^1^[37]. ^2^See text for details. ^3^Forward primer: GCCTCCACTCCCTGAAGAC, reverse primer: GCAAGTCCCAAGACACTGAGATC, probe: TCAGGAAGAAT [C/A]CCCG, primers and probe were designed according to the anti sense strand of the gene.

### Tag SNP genotyping

Tag SNPs were genotyped using genomic DNA extracted from blood. TaqMan^® ^Pre-Designed SNP genotyping assays as well as TaqMan^® ^Custom Made SNP genotyping assays (Applied Biosystems, Foster City, CA, USA) were used (table 2) according to the TaqMan Allelic Discrimination technology [35] on the ABI PRISM 7900HT Sequence Detection System (Applied Biosystems, Foster City, CA, USA).

### Promoter sequencing

Promoter SNPs rs35652124, rs6706649 and rs6721961 were genotyped by sequencing. We amplified a 423 bp region of the *NFE2L2 *promoter using forward primer 5'-GACCACTCTCCGACCTAAAGG-3' and reverse primer 5'-CGAGATAAAGAGTTGTTTGCGAA-3', annealing temperature 59°C and 34 cycles on a PTC-200 ThermalCycler (Biorad, Hercules, CA, USA). The product was purified using Illustra™ GFX™ PCR Purification Kit (GE Healthcare, Little Chalfont, Buckinghamshire, UK). Sequence reactions were performed using BigDye v3.1 (Applied Biosystems, Forster City, CA, USA) and analyzed on an ABI PRISM 3100 Automated Sequencer (Applied Biosystems, Forster City, CA, USA). All sequence data were analyzed using the DNASTAR SeqMan^® ^software (DNASTAR Inc, Madison, WI, USA).

### Statistical Analyses

Demographics for the PD cases and controls were compared using χ^2^-statistics for categorical parameters, i.e. sex, family history of neurodegenerative disorders and smoking habits, and using Mann-Whitney U test for age at sampling. Effects of sex, family history of neurodegenerative disease and smoking habits were analyzed by identifying significantly relevant covariates using forward stepwise logistic or linear regression in each material.

All tag SNPs were analyzed for deviation from Hardy-Weinberg equilibrium using χ^2^-statistics. Single marker associations were performed using logistic or linear regression in an additive model (dd = 0, Dd = 1 and DD = 2, where D = minor allele and d = major allele).

Haplotype frequencies were estimated in the HelixTree 6.3 software using the expectation-maximization algorithm [36] yielding all possible haplotypes present in our materials. In subsequent analyses, however, only haplotypes with an overall estimated frequency of >1.0% were included, while the rarer haplotypes were pooled. In the regression analysis the phase uncertainty for each individual was taken care of by coding each haplotype covariate according to the phase probabilities.

The genes were analyzed to identify the haplotype window with the strongest association to PD diagnosis and AAO of the disease, and to identify the haplotype alleles responsible for the association. To this end, we used a sliding window approach with stepwise forward logistic or linear haplotype regression including relevant covariates. The impact of each associated haplotype allele of the identified window was then investigated with logistic or linear regression including relevant covariates. Pairwise LD between the individual tag SNPs and promoter SNPs was calculated according to Gabriel *et al *[34] by means of r^2^.

The p-value threshold for statistical significance used in this study was p = 0.05. To correct for multiple testing, Bonferroni correction for the number of studied SNPs was used in all single marker analyses and permutation tests with 10 000 permutations were performed in the sliding window model. Corrected p-values are designated as p_c_. The statistical softwares used were SYSTAT11 (SYSTAT Software GmbH, Erkrath, Germany) and HelixTree 6.3 (Golden Helix, Bozeman, MT, USA).

### Ethics

The study was approved by the regional ethics committee at the University of Gothenburg, Sweden, and the ethics committee of the Pomeranian Medical University, Szczecin, Poland and was in compliance with the Helsinki Declaration of 1975. Written informed consent was obtained from all subjects.

## Results

### Demographics

Swedish cases and controls were well matched in age. There were significant differences in the distribution of sex, family history of PD and smoking habits (table 1). We identified sex, family history of PD and ever smoking as significantly relevant covariates for the analyses of association with disease risk. Family history of PD alone was identified as a significant covariate in analyses of association with AAO.

Polish cases and controls were matched in sex. Age at sampling was significantly higher in controls then in PD cases (table 1). No covariates were identified as significantly relevant for the analysis of association with disease risk. We identified sex as a significant covariate for analysis of association with AAO.

### Tag SNP genotyping

None of the studied markers either in the Swedish or the Polish material had a Hardy-Weinberg equilibrium p-value of < 0.01. The call rate was >95% for both TaqMan genotyping and sequencing.

### Association analysis - Swedish material

After correction for multiple testing none of the tag SNPs alone significantly affected risk of PD (table 3). The A allele of tag SNP 6 (rs10183914) in *NFE2L2 *was estimated to increase AAO of PD with four years per allele (p_c _= 0.028). No associations were seen for the markers in *KEAP1 *with either risk of PD (table 3) or AAO in PD (data not shown).

**Table 3:** Single marker frequencies and associations with risk of PD

SNP^1^	rs-ID	Genotype	Swedish material			Polish material		
				
			Parkinson n = 165^2^	Control n = 190^2^	p-value^3^ (p_c_-value)	Parkinson n = 192^2^	Control n = 192^2^	p-value^3^ (p_c_-value)
***NFE2L2***								
1	rs16865105	GG	9 (5.5)	18 (9.5)	0.720	10 (5.5)	9 (4.9)	0.716
		GT	59 (36.0)	62 (32.6)		58 (32.0)	65 (35.5)	
		TT	96 (58.5)	110 (57.9)		113 (62.5)	109 (59.6)	
2	rs7557529	AA	39 (23.8)	56 (29.5)	0.782	60 (32.1)	49 (27.4)	**0.043**
		AG	85 (51.8)	92 (48.4)		90 (48.1)	75 (41.9)	(0.473)
		GG	40 (24.4)	42 (22.1)		37 (19.8)	55 (30.7)	
P1	rs35652124	GG	15 (9.3)	13 (7.1)	0.564	26 (13.6)	17 (9.1)	0.053
		AG	58 (35.8)	89 (48.3)		77 (40.3)	67 (35.8)	
		AA	89 (54.9)	82 (44.6)		88 (46.1)	103 (55.1)	
P2	rs6706649	AA	4 (2.5)	3 (1.6)	1.000	6 (3.1)	2 (1.1)	1.000
		AG	33 (20.4)	45 (24.5)		35 (18.3)	42 (22.5)	
		GG	125 (77.1)	136 (73.9)		150 (78.6)	143 (76.4)	
P3	rs6721961	AA	1 (0.6)	1 (0.5)	0.056	6 (3.1)	2 (1.1)	0.833
		AC	39 (24.1)	27 (14.7)		38 (19.9)	43 (23.0)	
		CC	122 (75.3)	156 (84.8)		147 (77.0)	142 (75.9)	
3	rs2886161	GG	16 (9.7)	15 (7.9)	0.578	27 (14.4)	17 (9.4)	0.0656
		AG	59 (35.8)	91 (47.9)		75 (40.1)	67 (37.0)	
		AA	90 (54.5)	84 (44.2)		85 (45.5)	97 (53.6)	
4	rs1806649	AA	14 (8.5)	15 (7.9)	0.819	9 (4.8)	23 (12.7)	**0.011**
		AG	65 (39.4)	80 (42.1)		60 (32.3)	61 (33.7)	(0.121)
		GG	86 (52.1)	95 (50.0)		117 (62.9)	97 (53.6)	
5	rs2001350	GG	1 (0.6)	0 (0.0)	**0.016**	4 (2.1)	0 (0.0)	0.663
		AG	35 (21.2)	23 (12.1)	(0.224)	28 (14.7)	39 (20.9)	
		AA	129 (78.2)	167 (87.9)		158 (83.2)	148 (79.1)	
6	rs10183914	AA	14 (8.5)	25 (13.2)	0.111	20 (10.8)	28 (15.0)	0.254
		AG	81 (49.1)	89 (46.8)		73 (39.2)	73 (39.0)	
		GG	70 (42.4)	76 (40.0)		93 (50.0)	86 (46.0)	
7	rs2706110	AA	6 (3.7)	4 (2.1)	0.238	7 (4.0)	1 (0.6)	0.780
		AG	48 (29.3)	40 (21.1)		45 (25.4)	53 (30.6)	
		GG	110 (67.1)	146 (76.8)		125 (70.6)	119 (68.8)	
8	rs13035806	TT	2 (1.2)	2 (1.1)	0.796	2 (1.2)	0 (0.0)	0.323
		CT	29 (17.7)	27 (14.2)		28 (16.7)	27 (14.9)	
		CC	133 (81.1)	161 (84.7)		138 (82.1)	154 (85.1)	
***KEAP1***								
1	rs1048287	CC	1 (0.6)	2 (1.1)	0.589	---	---	---
		CT	35 (21.5)	42 (22.1)		---	---	
		TT	127 (77.9)	146 (76.8)		---	---	
2	rs11085735	TT	2 (1.2)	2 (1.1)	0.737	---	---	---
		GT	19 (11.7)	24 (12.8)		---	---	
		GG	142 (87.0)	162 (86.1)		---	---	
3	rs1048290	GG	22 (13.5)	31 (16.3)	0.372	---	---	---
		CG	77 (47.2)	89 (46.9)		---	---	
		CC	64 (39.3)	70 (36.8)		---	---	

Data are presented as absolute numbers (percentages). ^1^For SNP locations see table 2. ^2^Genotype information available for most cases as specified by the n numbers. ^3^p-values were calculated using an additive logistic regression model including statistically relevant covariates.

The haplotype window of *NFE2L2 *consisting of the five consecutive tag SNPs 2-6 (rs7557529, rs2886161, rs1806649, rs2001350 and rs10183914) was strongly associated with risk of PD (p_c _= 0.008), as well as with AAO of the disease (p_c _= 0.003). Phasing of this window resulted in six haplotypes with a frequency of ≥ 5% in the PD group (table 4). Within this window, the haplotypes GAGGG and GAAAG were both associated with increased risk of PD, with an odds ratio of 2.4 (p = 0.007) and 3.7 (p = 0.010) per haplotype allele, respectively (table 5). Additionally, the haplotype GAAAA was estimated to increase AAO of PD with approximately five years per haplotype allele (p = 7 × 10^-4^, table 5).

**Table 4:** Haplotype frequencies in PD cases and controls

Haplotypes^1^	Swedish material		Polish material	
		
	Parkinson	Control	Parkinson	Control
***NFE2L2 tag SNPs 2-6***				
AGGAG	26.2%	30.6%	33.9%	28.2%
AAGAG	22.2%	22.0%	20.5%	20.5%
GAAAA	21.5%	26.7%	18.4%	27.7%
GAGGG	10.9%	5.5%	9.7%	9.6%
GAGAA	10.2%	9.1%	9.9%	7.1%
GAAAG	5.4%	1.7%	1.4%	2.5%
***NFE2L2 promoter P1-P3***				
AGC	47.5%	47.0%	40.8%	48.1%
GGC	27.2%	31.2%	33.8%	27.0%
AAC	12.6%	13.8%	12.3%	12.3%
AGA	12.6%	7.9%	13.1%	12.6%
***KEAP1 tag SNPs 1-3***				
TGC	55.8%	52.7%	---	---
TGG	26.1%	27.7%	---	---
CGG	11.0%	12.0%	---	---
TTC	6.7%	7.4%	---	---

Data are presented for haplotypes with frequencies ≥ 5.0% estimated with the EM-algorithm.^1^For rs-numbers see table 2.

**Table 5:** Summary of NFE2L2 haplotypes that influence risk of PD

*NFE2L2 *Haplotypes^1^	Swedish material		Swedish material		Polish material	
			
	Disease Risk (OR/allele)^2^	p-value^3^	AAO (Years/allele)	p-value^3^	Disease risk (OR/allele)^2^	p-value^3^
***Tag SNPs 2-6***						
GAAAA	0.9 (0.6-1.3)	0.450	+4.8	**7 × 10^-4^**	0.6 (0.4-0.9)	**0.005**
GAGGG	2.4 (1.2-4.5)	**0.007**	-0.1	0.952	0.9 (0.5-1.5)	0.699
GAAAG	3.7 (1.3-10.6)	**0.010**	-3.3	0.236	0.5 (0.2-1.6)	0.239
***Promoter P1-P3***						
AGC	0.9 (0.7-1.3)	0.538	+2.1	0.061	0.6 (0.4-0.9)	**0.003**
***Full haplotypes*^4^**						
G**AGC**AAAA	0.9 (0.6-1.4)	0.631	+4.6	**0.001**	0.4 (0.3-0.6)	**2 × 10^-6^**
G**AGA**AGGG	2.8 (1.4-5.5)	**0.002**	-0.3	0.886	0.5 (0.3-1.0)	**0.031**
G**AGC**AAAG	3.3 (1.1-9.7)	**0.028**	-2.5	0.387	0.4 (0.1-1.4)	0.150

^1^For rs-numbers see table 2. ^2^Data are given as OR (95% confidence interval). ^3^p values are calculated with logistic or linear regression models including the associated haplotypes and significantly relevant covariates in the specified windows. ^4^tag SNPs 2-6 and promoter SNPs P1-P3 in order according to chromosomal location (figure 1) with the promoter SNPs highlighted in bold.

With regards to *KEAP1*, phasing of all three tag SNPs (rs1048290, rs11085735 and rs1048287) resulted in four haplotypes with a frequency of ≥ 5% (table 4) without significant associations with risk or AAO of the disease (data not shown).

### Promoter analysis - Swedish material

SNPs in the *NFE2L2 *promoter (figure 1) have previously been shown to affect promoter activity and Nrf2 expression *in vitro *[37]. To test the hypothesis that the observed haplotype associations described above may be explained by linkage disequilibrium to these functional polymorphisms, we genotyped all individuals for three SNPs in the *NFE2L2 *promoter region by sequencing.

Phasing of the promoter window (rs35652124, rs6706649 and rs6721961) resulted in four haplotypes with a frequency of ≥ 5% (table 4). The AGA haplotype with low promoter activity [37] showed tendency to association with increased risk of PD (p = 0.056, table 5) and was in LD (r^2 ^= 0.53) with the risk haplotype GAGGG. The protective, disease-delaying haplotype GAAAA was in LD (r^2 ^= 0.39) with the promoter haplotype AGC, i.e. the common promoter variant associated with full Nrf2 expression. The promoter SNPs are located between tag SNPs 2 and 3 (figure 1) and analysis of the full risk haplotype G**AGA**AGGG (freq: PD = 10.8%, freq controls = 4.9%) was associated with risk of PD (p = 0.002) with an odds ratio for PD of 2.8 per haplotype allele (table 5). The full protective haplotype G**AGC**AAAA (freq: PD = 22.5%, freq controls = 26.4%) was estimated to delay AAO in PD (p = 0.001) with approximately 5 years per haplotype allele (table 5).

### Replication of NFE2L2 associations - Polish material

In line with the results from the Swedish material, none of the tag SNPs or promoter SNPs alone had a significant effect on the risk of PD after correction for multiple testing (table 3). The observed association of rs10183914 with AAO in the Swedish material could not be replicated (data not shown).

In line with the results from the Swedish material, the haplotype window consisting of tag SNPs 2-6 was associated with risk of PD (p = 0.005). The haplotype GAAAA of this window was associated with decreased risk of PD (p = 0.005) with an odds ratio of 0.6 (table 5). Notably, this haplotype is identical to the protective haplotype associated with delayed AAO in the Swedish material. As in the Swedish material, GAAAA was in LD (r^2 ^= 0.42) with the common promoter haplotype AGC. Furthermore, the AGC haplotype alone showed association with reduced risk of PD (p = 0.003) with an odds ratio of 0.6 per haplotype allele (table 5). The full protective haplotype G**AGC**AAAA (freq: PD = 12.6%, controls = 27.8%) showed strong association with risk of PD (p = 2 × 10^-6^) with an odds ratio for PD of 0.4 for heterozygous carriers (table 5) and 0.2 (95% CI 0.1-0.4) for homozygous carriers. We could not replicate the risk association of the full haplotype G**AGA**AGGG (freq: PD = 5.9%, controls = 9.6%), since this haplotype showed association with reduced, rather than increased, risk of PD (p = 0.031) in the Polish material. There was no association with AAO in the Polish material (data not shown).

## Discussion

To our knowledge, this is the first case-control haplotype study showing association of the Nrf2-encoding *NFE2L2 *gene with a neurodegenerative disease. In *NFE2L2*, we found a region, including the promoter, which was clearly associated with risk of PD in two independent case-control materials. A haplotype including the fully functional variant of the promoter (G**AGC**AAAA) was associated with delayed AAO in the Swedish material and reduced risk of PD in the Polish material. These results support each other and are in agreement with data from animal and *in vitro *models suggesting important protective functions of Nrf2 in the central nervous system [22,23,25], and more specifically, in the nigrostriatal dopaminergic pathway affected in PD [27].

SNPs in *NFE2L2 *have previously been investigated for association with PD in two data sets for which data is released to the public. The first was a Japanese multiple candidate gene study [28]. This study included three *NFE2L2 *SNPs: rs2886161, rs2886162 and rs2706112, which showed no evidence of single marker associations with PD. The other study was an American two tiered whole-genome association study (first: sib pair, second: case-control) [38]. This study included six *NFE2L2 *SNPs (rs2706110, rs10183914, rs6726395, rs34820876, rs13005431, and rs6433657) of which none were included in our study. The SNP rs6726395 showed association with PD (p = 9 × 10^-3^) in the first tier of their study but was not replicated in the second tier (p = 0.9). This SNP is in LD (r^2 ^= 0.9) with rs7557529 that in our study showed association with risk of PD in the Polish material (p = 0.04), but not in the Swedish (p = 0.8).

The haplotype block identified in the Swedish discovery material consisted of five consecutive tag SNPs that are located upstream of or in intronic regions of *NFE2L2*. The disease-associated haplotypes could thus influence expression of Nrf2 or be linked to non-synonymous SNPs. However, SNPs that affect transcription factor function are particularly rare [39] and there are no non-synonymous SNPs with a reported frequency ≥ 5% within *NFE2L2 *either in HapMap or in the NCBI SNP database (dbSNP). In addition, the gene and protein sequences are >80% conserved across mammalian species, supporting a strong selection pressure against genetic variation in coding sequences of *NFE2L2. NFE2L2 *promoter polymorphisms, on the other hand, have previously been studied and found to affect *NFE2L2 *promoter activity *in vitro *[37]. Analysis of the promoter haplotypes in our materials suggests that part of the haplotype associations with risk of PD is explained by linkage to these functional promoter SNPs. Indeed, the protective haplotype GAAAA is in linkage with the wild type, well functioning version of the promoter AGC, and the full AGC-including haplotype G**AGC**AAAA was associated with decreased risk of PD in the Polish material and older AAO of PD in the Swedish material.

While an AAO-modifying gene is conceptually not the same as a risk gene they represent two overlapping concepts. An AAO-modifying gene will easily appear as a risk gene in certain study designs. The two studies differ substantially in the PD patients' AAO. The Swedish patients were on average 4 years older at disease onset compared with Polish patients. Depending on the shape of the age-dependent penetrans function for the *NFE2L2 *haplotypes, the Polish material may be better suited for detecting risk associations, while it may be easier do detect a genetic influence on AAO in the Swedish material.

In the Polish material the significant association was as the protective GAAAA haplotype while in the Swedish the GAGGG haplotype was associated with increased risk. For obvious reasons the presence of a risk/protective haplotype implies that other haplotypes must be protective/risk haplotypes. Since the different haplotypes viewed as covariates are negative confounders (due to the constraint of a total sum of two haplotypes per individual) it is a statistical phenomenon that the regression analysis can find significance for a protective haplotype in one material, while in another material a risk haplotype is found significant.

*KEAP1 *showed no association with PD in the Swedish or the Polish material. This finding is consistent with the American genome-wide study discussed above in which three *KEAP1 *SNPs (rs11085735, rs1048287, and rs2007529) were included but did not show any association with PD [38].

## Conclusions

In summary, a common *NFE2L2 *haplotype influences risk of PD in two discrete Caucasian case-control materials. The molecular consequence of this haplotype may be increased efficiency in the Keap1-Nrf2-ARE response to oxidative stress and thereby higher capacity to withstand endogenous or environmental risk factors for PD. Further investigations in other populations as well as functional studies addressing how the disease-associated *NFE2L2 *haplotype affects gene expression are now needed. To conclude, these results together with recent preclinical data provide another link between oxidative stress and the pathogenesis of PD and support *NFE2L2 *as a novel susceptibility gene for PD.

## Competing interests

The authors declare that they have no competing interests.

## Authors' contributions

**MVO**: Research project - Organization, design; Statistical analysis - Design and execution; Manuscript - Writing of first draft, review and critique. **SL**: Research project - Organization, design; Statistical analysis - Design and execution; Manuscript - Review and critique. **SN**: Statistical analysis - Design, review and critique; Manuscript - Review and critique. **DC**: Laboratory work - Execution; Manuscript - Review and critique. **PB**: Laboratory work - Execution; Manuscript - Review and critique. **AH**: Laboratory work - Supplier of patient material; Manuscript - Review and critique. **KB**: Research project - Conception; Manuscript - Review and critique. **MD**: Laboratory work - Supplier of patient material; Manuscript - Review and critique. **MB**: Laboratory work - Supplier of patient material; Manuscript - Review and critique. **MK**: Laboratory work - Supplier of patient material; Manuscript - Review and critique. **HN**: Laboratory work - Supplier of patient material; Manuscript - Review and critique. **MN**: Research project - Conception; Manuscript - Review and critique. **OH**: Research project - Conception; Manuscript - Review and critique. **HZ**: Research project - Conception; Manuscript - Writing of first draft, review and critique. All authors have read and approved the final manuscript.

## Pre-publication history

The pre-publication history for this paper can be accessed here:

http://www.biomedcentral.com/1471-2350/11/36/prepub

## References

[B1] Zhou C Huang Y Przedborski S Oxidative stress in Parkinson's disease: a mechanism of pathogenic and therapeutic significance Ann N Y Acad Sci 2008 1147 93 104 19076434 10.1196/annals.1427.023 PMC2745097

[B2] Kedar NP Can we prevent Parkinson's and Alzheimer's disease? J Postgrad Med 2003 49 3 236 245 14597787

[B3] Nunomura A Moreira PI Lee HG Zhu X Castellani RJ Smith MA Perry G Neuronal death and survival under oxidative stress in Alzheimer and Parkinson diseases CNS Neurol Disord Drug Targets 2007 6 6 411 423 10.2174/187152707783399201 18220780

[B4] Chinta SJ Andersen JK Redox imbalance in Parkinson's disease Biochim Biophys Acta 2008 1780 11 1362 1367 18358848 10.1016/j.bbagen.2008.02.005 PMC2547405

[B5] Andersen PM Amyotrophic lateral sclerosis associated with mutations in the CuZn superoxide dismutase gene Curr Neurol Neurosci Rep 2006 6 1 37 46 10.1007/s11910-996-0008-9 16469270

[B6] Bonifati V Rizzu P van Baren MJ Schaap O Breedveld GJ Krieger E Dekker MC Squitieri F Ibanez P Joosse M van Dongen JW Vanacore N van Swieten JC Brice A Meco G van Duijn CM Oostra BA Heutink P Mutations in the DJ-1 gene associated with autosomal recessive early-onset parkinsonism Science 2003 299 5604 256 259 10.1126/science.1077209 12446870

[B7] Kensler TW Wakabayashi N Biswal S Cell survival responses to environmental stresses via the Keap1-Nrf2-ARE pathway Annu Rev Pharmacol Toxicol 2007 47 89 116 10.1146/annurev.pharmtox.46.120604.141046 16968214

[B8] Dhakshinamoorthy S Jaiswal AK Functional characterization and role of INrf2 in antioxidant response element-mediated expression and antioxidant induction of NAD(P)H:quinone oxidoreductase1 gene Oncogene 2001 20 29 3906 3917 10.1038/sj.onc.1204506 11439354

[B9] Itoh K Wakabayashi N Katoh Y Ishii T Igarashi K Engel JD Yamamoto M Keap1 represses nuclear activation of antioxidant responsive elements by Nrf2 through binding to the amino-terminal Neh2 domain Genes Dev 1999 13 1 76 86 10.1101/gad.13.1.76 9887101 PMC316370

[B10] Cullinan SB Gordan JD Jin J Harper JW Diehl JA The Keap1-BTB protein is an adaptor that bridges Nrf2 to a Cul3-based E3 ligase: oxidative stress sensing by a Cul3-Keap1 ligase Mol Cell Biol 2004 24 19 8477 8486 10.1128/MCB.24.19.8477-8486.2004 15367669 PMC516753

[B11] Furukawa M Xiong Y BTB protein Keap1 targets antioxidant transcription factor Nrf2 for ubiquitination by the Cullin 3-Roc1 ligase Mol Cell Biol 2005 25 1 162 171 10.1128/MCB.25.1.162-171.2005 15601839 PMC538799

[B12] Kobayashi A Kang MI Okawa H Ohtsuji M Zenke Y Chiba T Igarashi K Yamamoto M Oxidative stress sensor Keap1 functions as an adaptor for Cul3-based E3 ligase to regulate proteasomal degradation of Nrf2 Mol Cell Biol 2004 24 16 7130 7139 10.1128/MCB.24.16.7130-7139.2004 15282312 PMC479737

[B13] Zhang DD Lo SC Cross JV Templeton DJ Hannink M Keap1 is a redox-regulated substrate adaptor protein for a Cul3-dependent ubiquitin ligase complex Mol Cell Biol 2004 24 24 10941 10953 10.1128/MCB.24.24.10941-10953.2004 15572695 PMC533977

[B14] Kobayashi A Kang MI Watai Y Tong KI Shibata T Uchida K Yamamoto M Oxidative and electrophilic stresses activate Nrf2 through inhibition of ubiquitination activity of Keap1 Mol Cell Biol 2006 26 1 221 229 10.1128/MCB.26.1.221-229.2006 16354693 PMC1317630

[B15] Itoh K Chiba T Takahashi S Ishii T Igarashi K Katoh Y Oyake T Hayashi N Satoh K Hatayama I Yamamoto M Nabeshima Y An Nrf2/small Maf heterodimer mediates the induction of phase II detoxifying enzyme genes through antioxidant response elements Biochem Biophys Res Commun 1997 236 2 313 322 10.1006/bbrc.1997.6943 9240432

[B16] Wild AC Moinova HR Mulcahy RT Regulation of gamma-glutamylcysteine synthetase subunit gene expression by the transcription factor Nrf2 J Biol Chem 1999 274 47 33627 33636 10.1074/jbc.274.47.33627 10559251

[B17] Venugopal R Jaiswal AK Nrf1 and Nrf2 positively and c-Fos and Fra1 negatively regulate the human antioxidant response element-mediated expression of NAD(P)H:quinone oxidoreductase1 gene Proc Natl Acad Sci USA 1996 93 25 14960 14965 10.1073/pnas.93.25.14960 8962164 PMC26245

[B18] Alam J Stewart D Touchard C Boinapally S Choi AM Cook JL Nrf2, a Cap'n'Collar transcription factor, regulates induction of the heme oxygenase-1 gene J Biol Chem 1999 274 37 26071 26078 10.1074/jbc.274.37.26071 10473555

[B19] Vargas MR Pehar M Cassina P Beckman JS Barbeito L Increased glutathione biosynthesis by Nrf2 activation in astrocytes prevents p75NTR-dependent motor neuron apoptosis J Neurochem 2006 97 3 687 696 10.1111/j.1471-4159.2006.03742.x 16524372

[B20] Kraft AD Johnson DA Johnson JA Nuclear factor E2-related factor 2-dependent antioxidant response element activation by tert-butylhydroquinone and sulforaphane occurring preferentially in astrocytes conditions neurons against oxidative insult J Neurosci 2004 24 5 1101 1112 10.1523/JNEUROSCI.3817-03.2004 14762128 PMC6793572

[B21] Shih AY Johnson DA Wong G Kraft AD Jiang L Erb H Johnson JA Murphy TH Coordinate regulation of glutathione biosynthesis and release by Nrf2-expressing glia potently protects neurons from oxidative stress J Neurosci 2003 23 8 3394 3406 12716947 10.1523/JNEUROSCI.23-08-03394.2003 PMC6742304

[B22] Calkins MJ Jakel RJ Johnson DA Chan K Kan YW Johnson JA Protection from mitochondrial complex II inhibition in vitro and in vivo by Nrf2-mediated transcription Proc Natl Acad Sci USA 2005 102 1 244 249 10.1073/pnas.0408487101 15611470 PMC538748

[B23] Vargas MR Johnson DA Sirkis DW Messing A Johnson JA Nrf2 activation in astrocytes protects against neurodegeneration in mouse models of familial amyotrophic lateral sclerosis J Neurosci 2008 28 50 13574 13581 10.1523/JNEUROSCI.4099-08.2008 19074031 PMC2866507

[B24] Ramsey CP Glass CA Montgomery MB Lindl KA Ritson GP Chia LA Hamilton RL Chu CT Jordan-Sciutto KL Expression of Nrf2 in neurodegenerative diseases J Neuropathol Exp Neurol 2007 66 1 75 85 10.1097/nen.0b013e31802d6da9 17204939 PMC2253896

[B25] Siebert A Desai V Chandrasekaran K Fiskum G Jafri MS Nrf2 activators provide neuroprotection against 6-hydroxydopamine toxicity in rat organotypic nigrostriatal cocultures J Neurosci Res 2009 87 7 1659 1669 10.1002/jnr.21975 19125416

[B26] Clements CM McNally RS Conti BJ Mak TW Ting JP DJ-1, a cancer- and Parkinson's disease-associated protein, stabilizes the antioxidant transcriptional master regulator Nrf2 Proc Natl Acad Sci USA 2006 103 41 15091 15096 10.1073/pnas.0607260103 17015834 PMC1586179

[B27] Chen PC Vargas MR Pani AK Smeyne RJ Johnson DA Kan YW Johnson JA Nrf2-mediated neuroprotection in the MPTP mouse model of Parkinson's disease: Critical role for the astrocyte Proc Natl Acad Sci USA 2009 106 8 2933 2938 10.1073/pnas.0813361106 19196989 PMC2650368

[B28] Mizuta I Satake W Nakabayashi Y Ito C Suzuki S Momose Y Nagai Y Oka A Inoko H Fukae J Saito Y Sawabe M Murayama S Yamamoto M Hattori N Murata M Toda T Multiple candidate gene analysis identifies alpha-synuclein as a susceptibility gene for sporadic Parkinson's disease Hum Mol Genet 2006 15 7 1151 1158 10.1093/hmg/ddl030 16500997

[B29] Daniel SE Lees AJ Parkinson's Disease Society Brain Bank, London: overview and research J Neural Transm Suppl 1993 39 165 172 8360656

[B30] Westerlund M Belin AC Anvret A Hakansson A Nissbrandt H Lind C Sydow O Olson L Galter D Cerebellar alpha-synuclein levels are decreased in Parkinson's disease and do not correlate with SNCA polymorphisms associated with disease in a Swedish material FASEB J 2008 22 10 3509 3514 10.1096/fj.08-110148 18606870

[B31] Bergman O Hakansson A Westberg L Nordenstrom K Carmine Belin A Sydow O Olson L Holmberg B Eriksson E Nissbrandt H PITX3 polymorphism is associated with early onset Parkinson's disease Neurobiol Aging 31 1 114 117 10.1016/j.neurobiolaging.2008.03.008 18420308

[B32] The HapMap Consortium The International HapMap Project Nature 2003 426 6968 789 796 10.1038/nature02168 14685227

[B33] Barrett JC Fry B Maller J Daly MJ Haploview: analysis and visualization of LD and haplotype maps Bioinformatics 2005 21 2 263 265 10.1093/bioinformatics/bth457 15297300

[B34] Gabriel SB Schaffner SF Nguyen H Moore JM Roy J Blumenstiel B Higgins J DeFelice M Lochner A Faggart M Liu-Cordero SN Rotimi C Adeyemo A Cooper R Ward R Lander ES Daly MJ Altshuler D The structure of haplotype blocks in the human genome Science 2002 296 5576 2225 2229 10.1126/science.1069424 12029063

[B35] Livak KJ Allelic discrimination using fluorogenic probes and the 5' nuclease assay Genet Anal 1999 14 5-6 143 149 10084106 10.1016/s1050-3862(98)00019-9

[B36] Excoffier L Slatkin M Maximum-likelihood estimation of molecular haplotype frequencies in a diploid population Mol Biol Evol 1995 12 5 921 927 7476138 10.1093/oxfordjournals.molbev.a040269

[B37] Marzec JM Christie JD Reddy SP Jedlicka AE Vuong H Lanken PN Aplenc R Yamamoto T Yamamoto M Cho HY Kleeberger SR Functional polymorphisms in the transcription factor NRF2 in humans increase the risk of acute lung injury FASEB J 2007 21 9 2237 2246 10.1096/fj.06-7759com 17384144

[B38] Maraganore DM de Andrade M Lesnick TG Strain KJ Farrer MJ Rocca WA Pant PV Frazer KA Cox DR Ballinger DG High-resolution whole-genome association study of Parkinson disease Am J Hum Genet 2005 77 5 685 693 10.1086/496902 16252231 PMC1271381

[B39] Ramensky V Bork P Sunyaev S Human non-synonymous SNPs: server and survey Nucleic Acids Res 2002 30 17 3894 3900 10.1093/nar/gkf493 12202775 PMC137415

